# The Role of Thrombocyte Parameters in Retinopathy of Prematurity Development

**DOI:** 10.1155/2022/7518533

**Published:** 2022-01-31

**Authors:** Dilek Özkaya

**Affiliations:** Department of Ophthalmology, Süleyman Demirel University, Faculty of Medicine, Isparta, Turkey

## Abstract

**Purpose:**

To investigate the role of thrombocyte parameters in retinopathy of prematurity (ROP) development.

**Materials and Methods:**

This retrospective study included 120 preterm infants in total. Group 1 was formed by infants who developed type-1 ROP and received treatment. Group 2 was formed by infants who developed ROP and were not treated for ROP. Infants who did not develop ROP and whose retinal vascularization was completed in their follow-up formed Group 3. Gestational age, birth weight, and genders of groups were recorded. Platelet (PLT) count, mean platelet volume (MPV), and platelet distribution width (PDW) values were obtained from complete blood count. Platelet mass index (PMI) was calculated by multiplying the PLT count by MPV value. Thrombocytopenia was defined as PLT count <150 × 1000/*μ*L. All parameters were compared between the groups.

**Results:**

There were 40 preterm infants in each group. The mean PLT count was 272.43 ± 122.67 in Group 1, 333.32 ± 133.06 in Group 2, and 310.03 ± 119.41 in Group 3. The difference in PLT count between the groups was not significant (*p*=0.094). Thrombocytopenia was observed in 25% of Group 1, 10% of Group 2, and 10% of Group 3 (*p*=0.095). No statistically significant difference was found in terms of MPV, PDW, and PMI values between the groups (*p*=0.102, *p*=0.097, and *p*=0.298, respectively).

**Conclusions:**

Although PLT count was lower and thrombocytopenia rate was higher in the type-1 ROP group, the differences were not found to be significant. Further prospective studies are required to evaluate the role of thrombocytes in ROP pathogenesis.

## 1. Introduction

Retinopathy of prematurity (ROP) is a multifactorial vasoproliferative disorder developing in the incompletely vascularized retina of preterm infants. ROP is such an important disease accounting for approximately 40% of all childhood blindness in the world. As neonatal care advances, the survival of low birth weight infants increases, and consequently, ROP incidence is rising [1, 2]. Immaturity and extreme oxygen application are the main risk factors determined for ROP. Besides these, the association between ROP and comorbidities such as respiratory distress syndrome, bronchopulmonary dysplasia, anemia, thrombocytopenia, blood transfusion, patent ductus arteriosus, necrotizing enterocolitis, bacterial and fungal sepsis, and intraventricular hemorrhage has been also signified [3].

The pathogenesis of ROP is thought to consist of two phases. The first phase involves vaso-obliteration secondary to the decrease of the angiogenic factors including vascular endothelial growth factor (VEGF) and insulin-like growth factor-1 (IGF-1) in response to postnatal hyperoxia. Retinal hypoxia developing due to an increase of metabolic demands of the avascular retina leads to increased levels of VEGF, and as a result, vasoproliferation constitutes the second phase [4].

Angiogenesis plays a key role in the pathologic process of ROP and is regulated by the discharge of pro- and antiangiogenic factors. The functions of thrombocytes (platelet = PLT) include regulation of angiogenesis except for hemostasis. Thrombocytes and megakaryocytes store both pro- and antiangiogenic mediators in *α*-granules. VEGF is one of the proangiogenic factors that contribute to vascular endothelial cell proliferation and has a critical role in the pathogenesis of ROP. The limitative effect of thrombocytes in pathological retinal neovascularization has been also reported [5–7]. While thrombocytopenia was found significantly associated with severe ROP in some studies [7–10], no significant difference was found in mean PLT counts in another study [11]. Mean platelet volume (MPV) is a parameter that shows platelet activity. The reactivity of large platelets is more than small platelets metabolically. MPV values have been also investigated in ROP development [11, 12]. Platelet mass index (PMI) is another parameter that has been studied for ROP development. Korkmaz et al. reported that MPI values were significantly higher in the laser photocoagulation group in the second phase of ROP [13]. We aimed to investigate the role of thrombocyte parameters including PLT count, presence of thrombocytopenia, MPV, platelet distribution width (PDW), and PMI in ROP development.

## 2. Materials and Methods

The present study included premature infants scanned for ROP between June 2014 and December 2018 in the Private Isparta Hospital Neonatal Intensive Care Unit. Inclusion criteria for the whole study groups included premature infants (gestational age at delivery <37 weeks): (1) with gestational age at delivery of ≤32 weeks, (2) birth weight of ≤1500 grams, (3) with a requirement for cardiopulmonary support following delivery at 32–37 weeks of gestational age, and (4) considered to be under risk in terms of ROP by the neonatologist's discretion. Premature infants whose follow-ups were not regular and data were missing in their files were excluded from the study for each group. We divided the premature infants into three groups. Infants who developed type-1 ROP and were treated with laser photocoagulation or intravitreal anti-VEGF injection formed Group 1. Infants who developed ROP and were not treated for ROP (spontaneous regression of stage 1 and stage 2 ROP) formed Group 2. Group 3 was formed by infants who did not develop ROP and whose retinal vascularization was completed in their follow-ups. Diagnosis and staging of ROP were determined according to the guidelines of the International Committee for the Classification of ROP (ICROP) [14]. Early Treatment for ROP (ETROP) trial criteria (type-1 ROP criteria) were used for treatment decisions. Zone-I, any stage ROP with plus disease; zone-I, stage-3 ROP with or without plus disease; and zone-II, stage-2 or 3 with plus disease are accepted as treatment indications. While intravitreal anti-VEGF injection was applied for zone-I ROP, laser photocoagulation was applied for zone-II ROP and ROP with peripheral pale [15].

Tropicamide 0.5% and phenylephrine 1.25% were dropped 3 times with an interval of 10 minutes for pupillary dilatation one hour before the examination. Proparacaine hydrochloride 0.5% was applied for topical anesthesia. After attaching the eye speculum, firstly the anterior segment was evaluated. Then, zone-I was examined with binocular indirect ophthalmoscope and 20 or 28 *D* lenses. For evaluation of zone-II and zone-III, scleral indentation was applied. Vital signs were monitored during the examination.

The data of patients were obtained retrospectively from electronic medical records. For all groups, gestational age, birth weight, and gender were recorded. PLT count, MPV, and PDW values were abstracted from the complete blood count. PMI was calculated by multiplying PLT count by MPV value (PMI = PLT count × MPV/10^3^). The most recent PLT count, MPV, and PDW values before ROP treatment were recorded for Group 1. For Groups 2 and 3, available values were recorded from the data of 38 weeks around postmenstrual age or hospital discharge, whichever is first. Thrombocytopenia was defined as PLT count <150 × 1000/*μ*L.

SPSS IBM version 22.0 for Microsoft Windows was used for statistical analyses. Shapiro–Wilk normality test was applied to identify the distribution of data. Data without normal distribution were stated as median (min-max), and data with normal distribution were stated as mean ± standard deviation (SD). A one-way ANOVA test was performed for the comparison of data with normal distribution between the groups. For comparing data without normal distribution, Kruskal–Wallis test was used. The Chi-square test was used to analyze the nominal data. *p* value <0.05 was considered statistically significant.

## 3. Results

In this retrospective study, each group consisted of 40 patients. Demographic characteristics of patients including gestational age, birth weight, and gender are presented in Table 1. There was a significant difference in terms of gestational age and birth weight between the groups (*p* < 0.001). As generally ROP treatment necessitates in more immature premature infants, this difference between the groups arises from this situation. The difference of gender between the groups was not statistically significant (*p* < 0.236).

The mean platelet count was 272.43 ± 122.67 in Group 1, 333.32 ± 133.06 in Group 2, and 310.03 ± 119.41 in Group 3. Although PLT count was lower in Group 1, there was no significant difference regarding PLT counts (*p*=0.094). Thrombocytopenia was observed in 25% of Group 1, 10% of Group 2, and 10% of Group 3. The association of thrombocytopenia between the groups was not statistically significant (*p*=0.095). The number and percentages of patients with thrombocytopenia in groups and *p* values are shown in Table 2.

The differences of MPV, PDW, and PMI values between the groups were not found statistically significant (*p*=0.102, *p*=0.097, and *p*=0.298, respectively). The comparison of PLT counts, MPV, PDW, and PMI values between the groups, and *p* values are presented in Table 3.

## 4. Discussion

The major finding of the current study is that ROP severity is generally not associated with platelet parameters in premature infants. Therefore, follow-up of platelet counts and other parameters may not be useful for ROP evaluation. However, our data should be cautiously interpreted since our design is cross-sectional without multiple assessments. These limitations also can limit clinical utility in the given context, and particularly, the effects of previous treatments including platelet administrations could not be specified. However, the present design provides a snapshot of the relationship between ROP and thrombocytopenia considering severity of the disease although the results were negative.

There are several limitations to our study. One of them is the small number of preterm infants in groups. Also, the differences in mean gestational age and birth weight between the groups were statistically significant. The data of patients were retrospectively collected from medical records. PLT counts and other parameters were abstracted from weekly routine tests and obtained from clinical indications which were not related to the study. Additionally, we did not take into account platelet transfusions and thrombocytopenia durations. The most recent PLT counts and other parameters before ROP treatment were recorded for Group 1. Available values were recorded from the data of 38 weeks around postmenstrual age or hospital discharge, approximately matched with the ROP treatment group's postmenstrual age, for Group 2 and 3. We exactly do not know the number of platelet transfusions or thrombocytopenia durations, but these limitations are valid for all groups.

Thrombocytopenia is a common hematologic disease in neonates and results in mortality and morbidity. The prevalence of thrombocytopenia is estimated to be 1–5% in all neonates, 12% in preterms, and 20–40% in infants followed up and treated in intensive care units. As gestational age and birth weight decreases, the incidence of thrombocytopenia increases [16, 17]. Besides the important roles of platelets in hemostasis and thrombosis, the modulator roles of platelets in other physiopathologic processes such as atherosclerosis, immunity, inflammation, and angiogenesis have been shown in various studies [18]. Even though platelets include both pro- and antiangiogenic molecules, platelets have been thought to promote angiogenesis by inducing chemotaxis, differentiation, and proliferation of endothelial cells and collection of progenitor cells to vascular injury sites [19].

The major regulator role of angiogenic regulatory proteins, including VEGF, has been demonstrated in many studies. Platelets store, carry, and transmit VEGF and IGF-1. Although it is not exactly understood how thrombocytopenia affects VEGF balance and phases of ROP, it is claimed that proliferative retinopathy occurs with an increase in endogenous production of IGF-1 by activation of retinal VEGF accumulation in ROP pathogenesis (second phase). Accordingly, thrombocytopenia may contribute to inadequate VEGF sequestration in the immature retina and afterward development or advancement of proliferative retinopathy [6]. Because of this association between thrombocytes, VEGF, and ROP, the role of thrombocytopenia has been investigated. Vinekar et al. searched the effect of thrombocytopenia in aggressive posterior ROP (APROP). They reported a significant difference between PLT counts and APROP. Firstly, they realized the spontaneous regression of APROP in an index sick case with thrombocytopenia (21.000/mm^3^), soon after the correction of thrombocytopenia (118.000/mm^3^) with platelet transfusions. Then, they analyzed the other APROP cases. They reviewed other 9 APROP cases and 21 control subjects. There was a significant difference in terms of PLT counts between APROP cases and control subjects (*p*=0.0002). They concluded as it is probable that low platelet levels may lead to the lack of delivering the optimum levels or inadequate clearance of extreme VEGF generated from the ischemic, peripheral retina in preterm infants with APROP [20].

PLT counts and related parameters are studied in both the first and second phases of ROP. There was not found any difference in many studies regarding PLT parameters in the first phase. So, it is hypothesized that platelets may be more active and lead to an increase of neovascularization in the second phase. Therefore, there are some studies that include both the first and second phases or only the second phase in literature. Our study also included data from the second phase. While the role of PLT counts and thrombocytopenia in the development of ROP has been investigated in some studies; the role of MPV, PDW, and PMI in the development of ROP has been searched in only a few studies. We analyzed not only PLT counts and thrombocytopenia but also MPV, PDW, and PMI values in our study. Generally, study groups are formed by type-1 ROP or ROP cases treated with laser, and control groups are formed by infants who did not develop ROP or infants with ROP regressed spontaneously in the other studies [7–11, 13, 21, 22]. We separated the cases that did not develop ROP and the cases whose ROP regressed spontaneously in our study. Type-1 ROP treated with laser or intravitreal anti-VEGF injection formed the third group; thus, we analyzed the data of 3 different groups differently from the other studies.

Lundgren et al. investigated postnatal characteristics in APROP development. Thrombocytopenia occurred in all APROP subjects in the first month and 6 of 9 APROP cases during ROP diagnosis. APROP cases had lower median PLT counts (90 ×  109/L) when compared with control cases (158 × 109/L) (*p* < 0.001) [22]. PLT counts, thrombocytopenia, and infections were searched for ROP development in another study. The authors found a statistically significant difference in the occurrence of thrombocytopenia (*p*=0.015), platelet counts before the diagnosis of ROP (*p*=0.008), and the presence of late-onset infection (*p*=0.007) [21]. Jensen et al. investigated the association between thrombocytopenia and type-1 ROP development. The latest PLT counts before laser for the study group and matched postmenstrual age for the control group were analyzed in this retrospective 1 : 1 matched case-control study. Median PLT counts were lower in the type-1 ROP cases, but the difference was not statistically significant (*p*=0.09). They concluded that thrombocytopenia was associated with type-1 ROP, primarily zone-I ROP [7]. There was no significant difference in terms of PLT counts between the groups in other studies [11, 12, 23, 24]. Similarly, although PLT counts were lower in the type-1 ROP group, the difference was not significant in our study (*p*=0.094). Thrombocytopenia occurred in 25% of Group 1 and 10% of Groups 2 and 3. However, the association of thrombocytopenia between the groups was not statistically significant (*p*=0.095).

MPV, which measures the average size of platelets, is a potential biomarker of platelet activity. Larger platelets are more active than smaller platelets metabolically and enzymatically. They contain more *α*-granules and have more prothrombotic activity [25]. It is suggested that activated and larger platelets influence the development of type-1 ROP. Tao et al. found a significant difference in terms of MPV between the cases with ROP required laser treatment and control groups (10.76 ± 1.27 fL, 10.04 ± 0.91 fL, *p*=0.0001). They found no significant difference regarding the mean platelet counts or MPV/platelet counts between the groups (*p*=0.4151, *p*=0.1460, respectively). They emphasized the association between elevated MPV and type-1 ROP [11]. In another study, MPV was found as 9.1 ± 2.0 fL in cases with type-1 ROP group and 9.4 ± 1.8 fL in cases without type-1 ROP. The difference of MPV between the groups was not statistically significant (*p*=0.61). They also found no significant difference in terms of PLT counts between the groups (*p*=0.78) [12]. Korkmaz et al. reported no statistically significant difference between the control and laser groups in terms of PLT counts and MPV values during the first stage of the disease (*p* > 0.05). While PLT counts were not statistically different (*p*=0.98), there was a significant difference between the control and laser groups in terms of MPV (*p*=0.001) during the second stage of ROP [23]. In our study, the difference in MPV values between the groups was not statistically significant (*p*=0.102).

PDW is an indicator evaluating the variation of platelet size. It is regarded as a distinctive feature of platelet morphology. PDW values are generally evaluated for differential diagnosis of thrombocytopenia [26]. PDW is not widely used for assessing the association with ROP. Ünsal et al. aimed to find a potential parameter for estimating the risk of ROP development in their studies. PDW was found as 15.8 in the non-ROP group and 15.9 in the ROP group. The difference in PDW was not statistically significant (*p*=0.973) [24]. Similarly, no significant difference was found in terms of PDW between the groups in our study (*p*=0.097).

PMI, which is associated with platelet functionality, is one of the thrombocyte parameters searched for ROP in a few studies. Korkmaz et al. found no significant difference when they compared PLT counts for any ROP phases (*p* > 0.05). While the difference of PMI between the control (2040 ± 1133) and laser photocoagulation groups (2070 ± 968) was not significant during the first phase of ROP, the difference of PMI between the control (3230 ± 1419) and laser photocoagulation groups (3710 ± 1308) during the second phase was statistically significant (*p* > 0.05 and *p* < 0.05, respectively). It was also reported that PMI is inversely proportional to platelet activity [13]. We found no significant difference between the groups regarding PMI in our study (*p*=0.298).

## 5. Conclusions

ROP is one of the leading causes of blindness in childhood. The investigations about the risk factors of ROP are still going on. Although PLT counts were lower and the number of cases with thrombocytopenia was higher in the type-1 ROP group, we found no significant difference regarding PLT counts and thrombocytopenia between the groups. There were also no significant differences in terms of MPV, PDW, and PMI values in our study. Additional prospective studies with a large number of serials are necessary to clarify the relationship between thrombocyte parameters and ROP.

## Figures and Tables

**Table 1:** Demographic characteristics of patients.

	Group 1 *n* = 40	Group 2 *n* = 40	Group 3 *n* = 40	*p* value
*Gestational Age (weeks)*				
Median (min-max)	26, 5 (24–32)	30, 0 (25–33)	32, 0 (27–32)	<0.001^*a*^
*Birth Weight (g)*				
Median (min-max)	925, 0 (430–1440)	1305, 0 (740–2370)	1540, 0 (930–2620)	<0.001^*a*^
*Gender*				
Female, *n* (%)	19 (47.5)	26 (65.0)	20 (50.0)	0.236^*b*^
Male, *n* (%)	21 (52.5)	14 (35.0)	20 (50.0)	

^*a*^Kruskal–Wallis test. ^*b*^Chi-square test.

**Table 2:** The number and percentages of patients with thrombocytopenia in groups and *p* value.

		Groups						*p* value
		Group 1		Group 2		Group 3		
		*n*	%	*n*	%	*n*	%
TP	−	30	75	36	90	36	90	0.095^*a*^
	+	10	25	4	10	4	10	

TP: thrombocytopenia, ^*a*^: Chi-square-test.

**Table 3:** The comparison of PLT counts, MPV, PDW, and PMI values between the groups.

	Group 1 *n* = 40 Mean ± SD	Group 2 *n* = 40 Mean ± SD	Group 3 *n* = 40 Mean ± SD	*p* value
PLT count (×1000/*μ*L)	272.43 ± 122.67	333.32 ± 133.06	310.03 ± 119.41	0.094^*a*^
MPV (fL)	9.12 ± 1.52	8.74 ± 0.81	9.25 ± 0.82	0.102^*a*^
PDW(fL)	18.31 ± 8.37	15.63 ± 3.07	17.32 ± 2.98	0.097^*a*^
PMI (fL/nL)	2523.54 ± 1035.21	2849.13 ± 1034.82	2836.17 ± 1064.34	0.298^*a*^

^*a*^: one-way ANOVA test.

## Data Availability

The datasets used and/or analyzed during the current study are available from the corresponding author on reasonable request.
